# A Proposed Simple and Accurate Technique for Optimal Long-Term Hemodialysis Catheter Tip Placement

**DOI:** 10.5334/jbsr.1474

**Published:** 2018-01-31

**Authors:** Sammy Tawk, Elie Barakat, Frank Hammer

**Affiliations:** 1 Cliniques univeristaires Saint-Luc, BE

**Keywords:** Catheter, dialysis, technique, interventional radiology

## Abstract

We describe a simple ultrasound (US)-guided technique for accurate anatomical right atrium localization prior to permanent hemodialysis catheter insertion. It is used in patients for whom a permanent hemodialysis catheter will be inserted through an internal jugular vein access, in order to have the functional catheter tip located at the mid-level of the right atrium. In this technique, the right atrium is localized on US via left intercostal approach prior to catheter insertion under fluoroscopic guidance.

## Introduction

Adequate vascular access is the *sine qua non* for successful hemodialysis in end-stage renal disease [1]. Native arteriovenous (AV) fistula is the preferred way of vascular access due to its multiple advantages over the others [2]. Unfortunately, in many circumstances where an AV fistula is either pending, not feasible or non-functional, another way of vascular access must be established. Long-term hemodialysis catheter can be instantly used for dialysis and is generally easily inserted by a well-trained interventional radiologist.

Optimal positioning of the functional catheter tip is crucial for efficient hemodialysis [3] and the recommended anatomical location has been long debated. The latest Kidney Foundation Kidney Disease Outcomes Quality Initiative (KDOQI) guidelines recommend placing the functional catheter tip in the right atrium (mid-level) with its arterial lumen facing the mediastinum [2] to prevent blood recirculation and to avoid catheter induced superior vena cava complications [1].

Multiple radiological landmarks have been proposed to localize the cavoatrial junction but none of them being so accurate due to the high interindividual anatomical variability [4,5,6].

To precisely locate the right atrium for optimal catheter tip positioning, a simple and accurate technique should be used.

We will describe a simple technique used in our department to precisely locate the right atrium for optimal catheter tip positioning.

## Technique

The right internal jugular vein is the first-line choice for catheter insertion. If contraindicated (maturing arteriovenous fistula at right or thrombosed vein), the left side is chosen. If both internal jugular veins are non-accessible, the femoral access can be used. It is always better to avoid the subclavian venous access in order to preserve an intact draining pathway for a potential AV fistula. The procedure is done under local anaesthesia and continuous monitoring. The right atrium localization is performed before draping the patient by using a low frequency convex probe or preferably a dedicated cardiac probe, a US machine being always available for the venous puncture.

By a left intercostal window, the four chambers of the heart are identified. Then, by swiping the US probe to a strictly horizontal plane (parallel to the x-ray beam that is perpendicular to the table during catheter insertion), the right atrial floor is localized, and if not possible, the atrial lumen in the horizontal plane should be localized. With the probe held in a fixed position, a landmark is put by drawing a horizontal line on the skin at the same level of the probe. Finally, a metallic landmark (paper clip) is stuck to the skin at right, at the same horizontal level to the drawn skin marker.

After right atrial localization, an estimation of the approximate catheter length from the skin entry site to the marker is performed with the infra-clavicular skin entry site modified accordingly. In adults, a 23 or 27 cm length split tip catheter is generally used whether the right or left internal jugular vein is chosen respectively.

**Figure 1:**
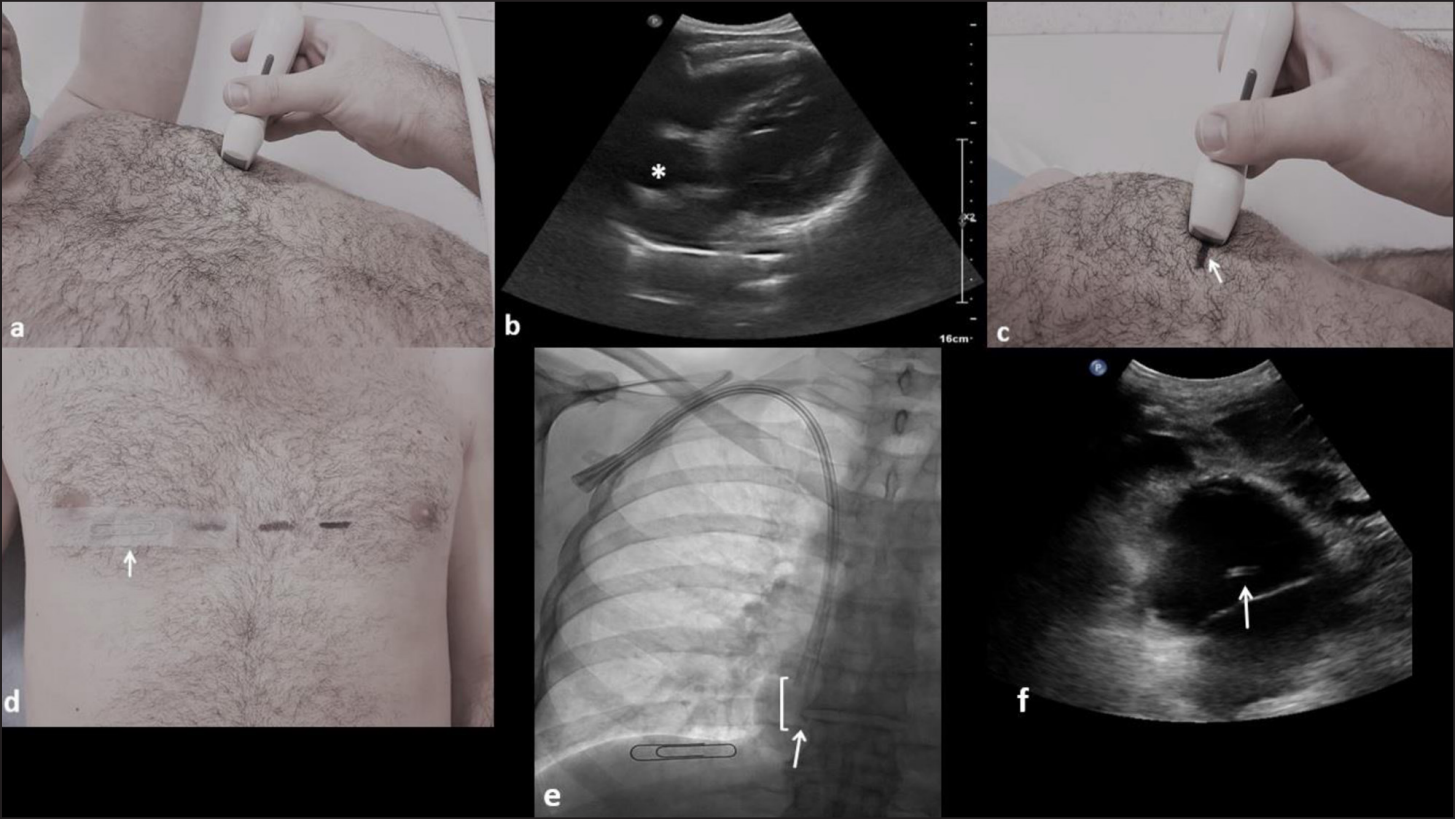
**(a)** The US probe is placed transversely in a left anterior intercostal space. **(b)** The right atrium is localized (asterisk) by a left intercostal approach. **(c)** A horizontal line is drawn on the skin (arrow) at the probe’s level (mid-width). **(d)** A paper clip (arrow) is stick on the skin at right (midclavicular line), at the same horizontal level that the drawn skin marker, corresponding to the atrial floor. **(e)** Antero-posterior fluoroscopic image of the chest showing a 23 cm inserted long term hemodialysis catheter through the right internal jugular vein with its distal tip (arrow) located above the paper clip, so the entire functional part (square bracket) is located inside the right atrium. **(f)** Subxiphoid US view confirming the distal catheter tip position inside the right atrium (arrow).

The catheter is then inserted under fluoroscopic guidance and finally fixed and sutured to the skin with its distal tip located few millimeters and up to 1.5 cm above the metallic landmark, so the entire functional part is located inside the right atrium. At the end of the procedure, a subxiphoid cardiac US view confirms the correct positioning of the catheter tip (Figure 1).

## Discussion

Vascular access in end stage renal disease is a cornerstone for therapy; it should be adequate to assure efficient hemodialysis [1]. There are many ways of vascular accesses that can be used for hemodialysis with AV fistulae being the first choice [2]. Unfortunately, maintaining a long-term functional vascular access is challenging [2] and sometimes, the only available way to perform hemodialysis is through venous catheter insertion when, for example, urgent dialysis is needed or while awaiting fistula maturation.

The distal tip position of the long term hemodialysis catheter is the mainstay for adequate functioning and subsequent efficient dialysis [3]. In fact, blood recirculation is an important limiting factor in theses catheters and manufactures are constantly working on different designs, trying to minimize this factor.

Another way to prevent recirculation is correct tip positioning [1]. Unfortunately, the optimal position has been long debated, and until 2006, the recommendations were to place the catheter tip in the superior vena cava to prevent cardiac perforations [7]. However, vessel wall injury, positional malfunctioning from venous wall suction and higher recirculation rates have all been associated with positioning the catheter tip at this level [8,9].

The latest KDOQI guidelines recommend positioning the catheter tip in the mid atrium with its arterial lumen facing the mediastinum [2] without reaching the atrial floor to avoid perforation, arrhythmias and mural thrombus formation [4,10].

Many anatomical landmarks have been proposed to precisely locate the cavoatrial junction on conventional radiography or fluoroscopy but none of them were very accurate or reproducible due to anatomical inter-individual variability [4,5,6].

Another technique for central venous catheter tip localization using US have also been proposed, either by direct visualization of the catheter tip or by using intravenous contrast-enhanced US [11]. The second technique is performed by injecting a prepared microbubble solution in the inserted catheter lumen while looking at the right atrium by a subcostal view. By analysing the time from injection to visualization of the microbubbles flow, an estimation of the catheter tip localization is done [11]. These two US techniques have been proven to accurately localize the catheter tip and prevent malposition of regular central venous catheters [12,13,14,15]. Their disadvantage, when used in long term hemodialysis catheter insertion, is that they are performed after the insertion of the catheter, thus, the exact catheter length and entry skin site cannot be determined in advance. In addition, these techniques are time consuming due to the fact that US localization and/or the intravenous contrast preparation/administration need to be done under strict aseptic condition to readjust the catheter if needed.

Our described technique has the advantage of being quickly performed before the patient has been draped so an estimation of the catheter length and the entry skin site can be obtained. Furthermore, no additional material or special training is required. Its limitations are mainly those of transthoracic echocardiography (i.e. body habitus, emphysema) where the right atrium cannot be identified.

Finally, concerning the possible upward migration when the patient is in an erect or semi-erect position, regardless of the performed technique, a shorter and lateral tunnel should be done to minimalize catheter tip shifting.

## Conclusion

Long term hemodialysis catheter insertion is a routine and quite simple procedure for every interventional radiologist. It should always be performed in the interventional radiology department under fluoroscopic and ultrasound guidance. Accurate identification of the right atrium is mandatory to correctly position the functional catheter tip at that level and ensure a long-lasting functional catheter.
